# Functional Subdivision of Group-ICA Results of fMRI Data Collected during Cinema Viewing

**DOI:** 10.1371/journal.pone.0042000

**Published:** 2012-07-30

**Authors:** Siina Pamilo, Sanna Malinen, Yevhen Hlushchuk, Mika Seppä, Pia Tikka, Riitta Hari

**Affiliations:** 1 Brain Research Unit, O.V. Lounasmaa Laboratory, School of Science, Aalto University, Espoo, Finland; 2 Advanced Magnetic Imaging Centre, O.V. Lounasmaa Laboratory, School of Science, Aalto University, Espoo, Finland; 3 Department of Motion Picture, Television and Production Design, School of Art, Design and Architecture, Aalto University, Helsinki, Finland; Wake Forest School of Medicine, United States of America

## Abstract

Independent component analysis (ICA) can unravel functional brain networks from functional magnetic resonance imaging (fMRI) data. The number of the estimated components affects both the spatial pattern of the identified networks and their time-course estimates. Here group-ICA was applied at four dimensionalities (10, 20, 40, and 58 components) to fMRI data collected from 15 subjects who viewed a 15-min silent film (“At land” by Maya Deren). We focused on the dorsal attention network, the default-mode network, and the sensorimotor network. The lowest dimensionalities demonstrated most prominent activity within the dorsal attention network, combined with the visual areas, and in the default-mode network; the sensorimotor network only appeared with ICA comprising at least 20 components. The results suggest that even very low-dimensional ICA can unravel the most prominent functionally-connected brain networks. However, increasing the number of components gives a more detailed picture and functionally feasible subdivision of the major networks. These results improve our understanding of the hierarchical subdivision of brain networks during viewing of a movie that provides continuous stimulation embedded in an attention-directing narrative.

## Introduction

Data-driven analysis methods, such as independent component analysis (ICA), are gaining increasing interest in providing reliable analyses of functional magnetic resonance imaging (fMRI) signals collected during naturalistic complex stimuli [1]–[4]. ICA can separate fMRI data into additive components that comprise spatially independent, functionally connected brain networks. However, no exact rules exist for estimating the correct number of independent components (ICs). Furthermore, previous investigations have come to partly controversial conclusions about the proper number of independent components, and especially how this number is related to the functional feasibility of the results [5]–[8].

When the dimensionality of the ICA is increased, the ICs typically split into subcomponents. Too low dimensionality can lead to loss of information [9] or to confusing mixtures of several components [2], [5], [10]–[12]. Thus it has been suggested that one should prefer high rather than low dimensionality [13]. However, the excess of components may decrease the stability and reliability of the IC estimates [5], [7].

A previous study [14] used a large fMRI database to compare the ICA results at dimensionalities of 20 and 70 components, focusing on the splitting of resting-state networks. However, ours is the first study on the relationship between the number of estimated ICs and the functional organization of brain networks during viewing a movie that closely resembles every-day life conditions and–in addition to the continuous and complex visual stimulation–provides attention-capturing narratives.

We used a 15-min long, skillfully directed silent film as a rich and continuous visual stimulus to study how the dimensionality of the ICA (10, 20, 40, or 58 components) affects the subdivision of three major functional brain networks: the dorsal attention network (DAN), the default-mode network (DMN), and the sensorimotor network (SMN). We were especially interested in the hierarchy of the networks and aimed to find out whether the networks would split into functionally feasible subunits when the model order is increased or whether the splitting is arbitrary at high dimensionalities. We started from the component count 58 suggested by the MDL method and selected three lower counts to observe the merging and splitting of these networks. Higher model orders were not studied here since the MDL method tends to already overestimate the number of components [15].

## Results

### Brain Networks at the Dimensionality of 10 ICs

**Figure 1:**
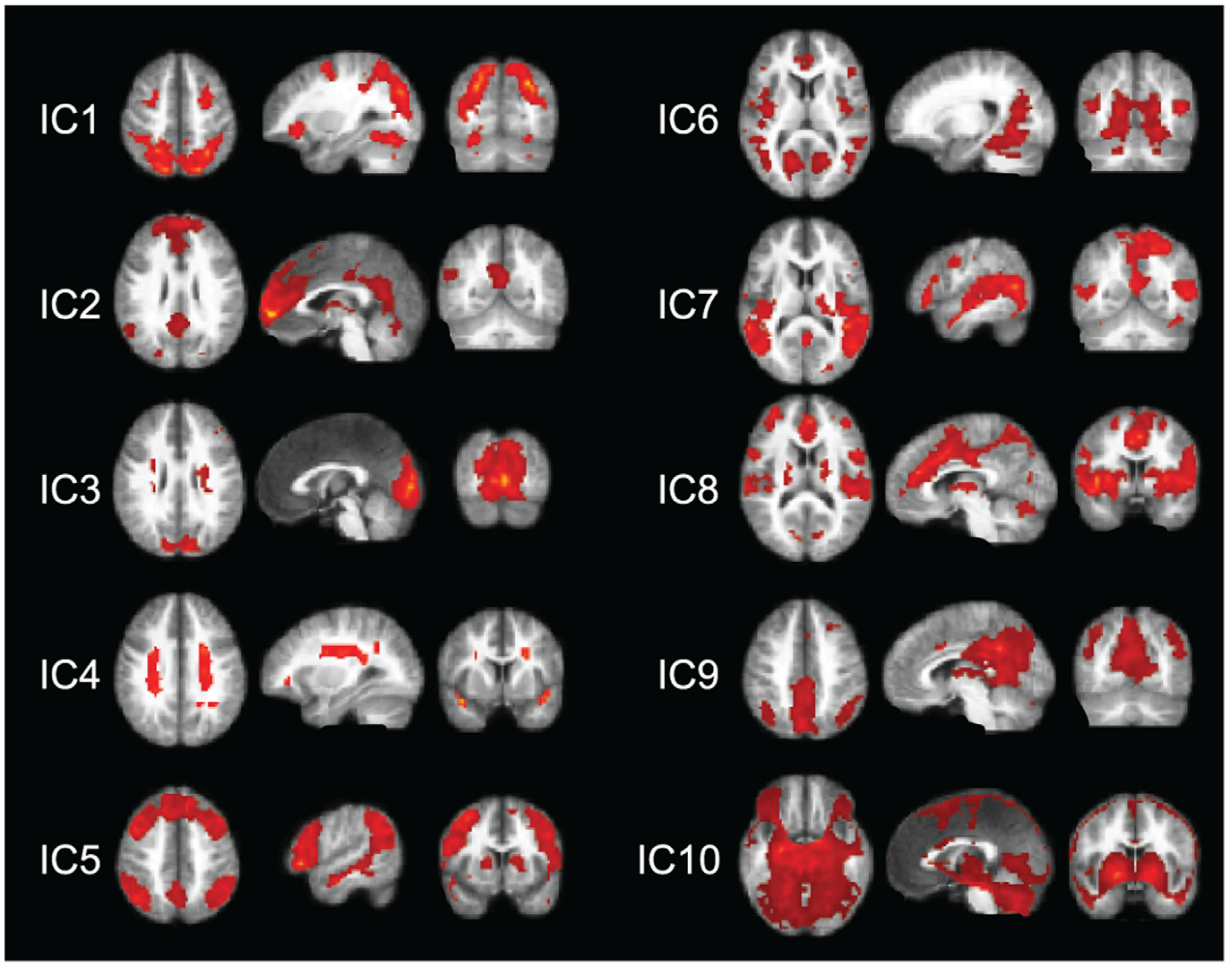
10-IC decomposition. Spatial maps for the 10 ICs of the 10-IC decomposition, each viewed in three orthogonal directions. The group-level t-maps are thresholded at *t ≥*6.

Figure 1 illustrates all components of the lowest-dimension (10-IC) decomposition. The components cover both the dorsal attention network DAN and the default-mode network DMN without any clear sign of the SMN. In this decomposition, the components covering the DAN and DMN also included other brain areas that are not typically listed to these networks.

IC1 captures the DAN, including the frontal eye fields (FEFs) and the intraparietal sulcus (IPS) bilaterally; the IPS activity extends down to the supramarginal gyri and the V5/MT region. Other prominent activities were seen in the fusiform gyri bilaterally, including the posterior part of the inferior temporal cortex, as well as in the middle frontal gyri bilaterally.

IC2 corresponds to the DMN, covering the medial prefrontal cortex, the posterior cingulate (PCC; also extending to the precuneus, preC), and the left inferior parietal cortex (IPC). It also includes activity in the midline cerebellar vermis, in the anterior insula and caudate bilaterally, as well as in left thalamus (only some of these areas visible in Fig. 1).

IC3 covers the striate and extrastriate visual cortices. It also contains elongate bilateral artifacts in the white matter, about 1.5 cm lateral to both lateral ventricles.

IC4 includes prominent, several centimeters long bilateral signal source–an apparent artifact–in the white matter, similar to those in IC3, as well as activity in the insular cortices.

IC5 covers the prefrontal cortex extensively, the IPC bilaterally and the preC. It also includes the superior temporal sulcus (STS)/upper part of the medial temporal gyrus along the whole length of the STS, as well as the caudate in both hemispheres, and the right anterolateral thalamus.

In IC6, the most prominent activity covers the inferior occipital cortex, mainly the lingual gyri, and the medial occipital lobe bilaterally. It also includes multiple locations in the STS, and left-hemisphere-dominant posterior insula, as well as nodes in the anterior cingulate, interior frontal gyri, amygdala/anterior hippocampus, and putamen in both hemispheres.

IC7 is quite patchy. The main node covers the top of the superior midline parietal lobe. It also includes the whole STS and middle and posterior STG extending to the posterior part to the MT/V5 region; both latter activations are bilateral but right-hemisphere dominant. It also covers the right thalamus, hippocampus, amygdala, and posterior insula.

In IC8, midline activation extends from the anterior to the middle cingulate cortex. It also contains a widespread bilateral activation in the superior temporal lobes, insulae, caudate, and cerebellum.

IC9 is dominated by a wide bilateral activation in the posterior cingulate cortex (extending to the top of the mesial posterior parietal cortex) and the inferior parietal lobe (focus on the angular gyrus), posterior to the IPS activity spot in the DMN component IC2. Activation is also seen in anterior and posterior thalamus.

IC10 captures an apparent artifact, comprising wide-spread patchy activations.

**Figure 2:**
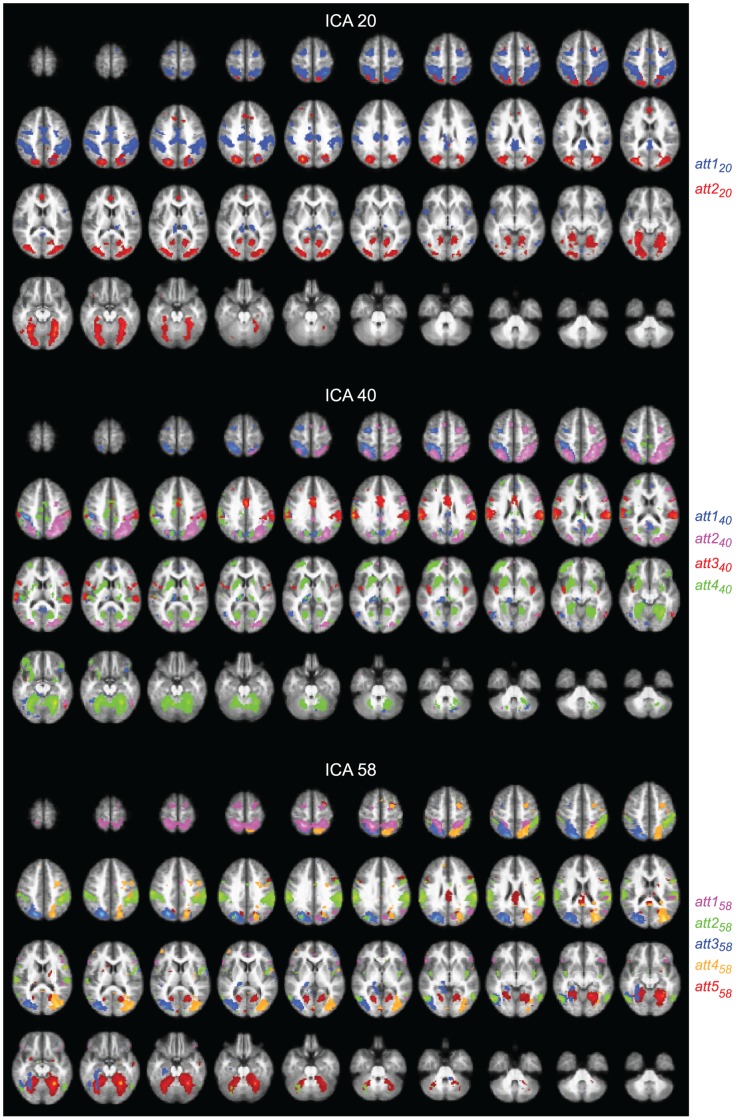
Dorsal attention network. Spatial maps for the ICs selected to represent the dorsal attention network (DAN) in the 20- (top), 40- (middle), and 58-IC (bottom) decompositions, with 2, 4, and 5 color-coded components displayed, respectively. The maps are thresholded at *t ≥*6.

**Figure 3:**
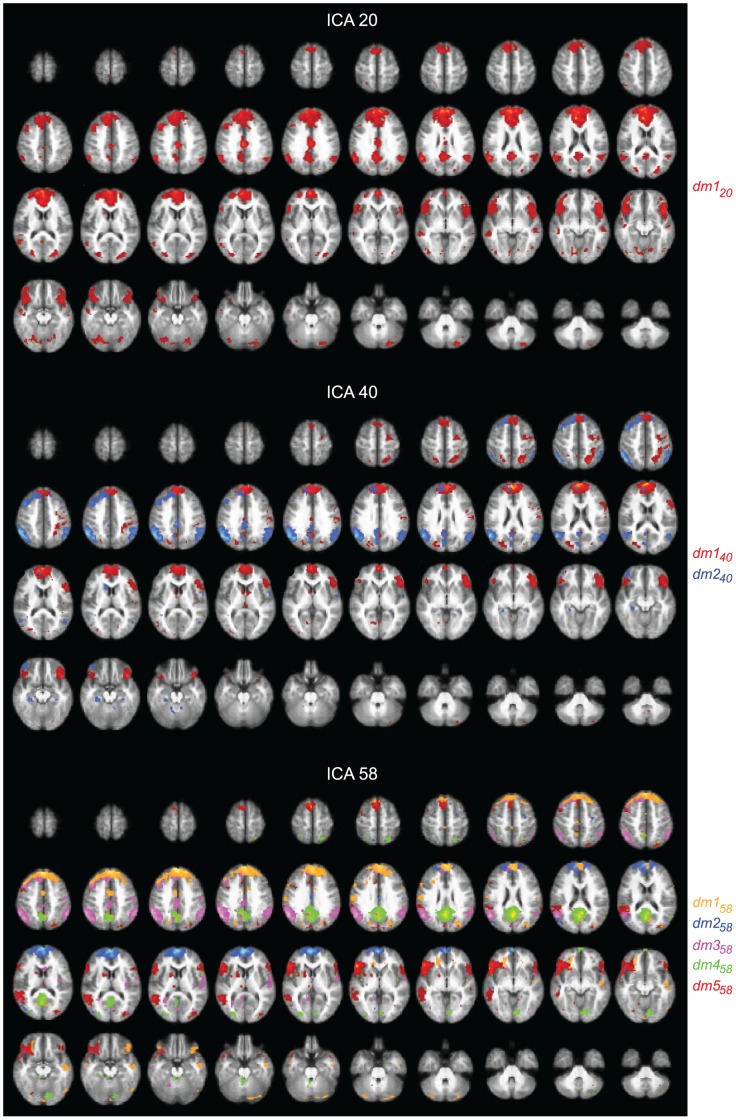
Default-mode network. Spatial maps for the ICs selected to represent the DMN in the 20- (top), 40- (middle), and 58-IC (bottom) decompositions, with 1, 2, and 5 color-coded components displayed, respectively. The maps are thresholded at *t ≥*6.

**Figure 4:**
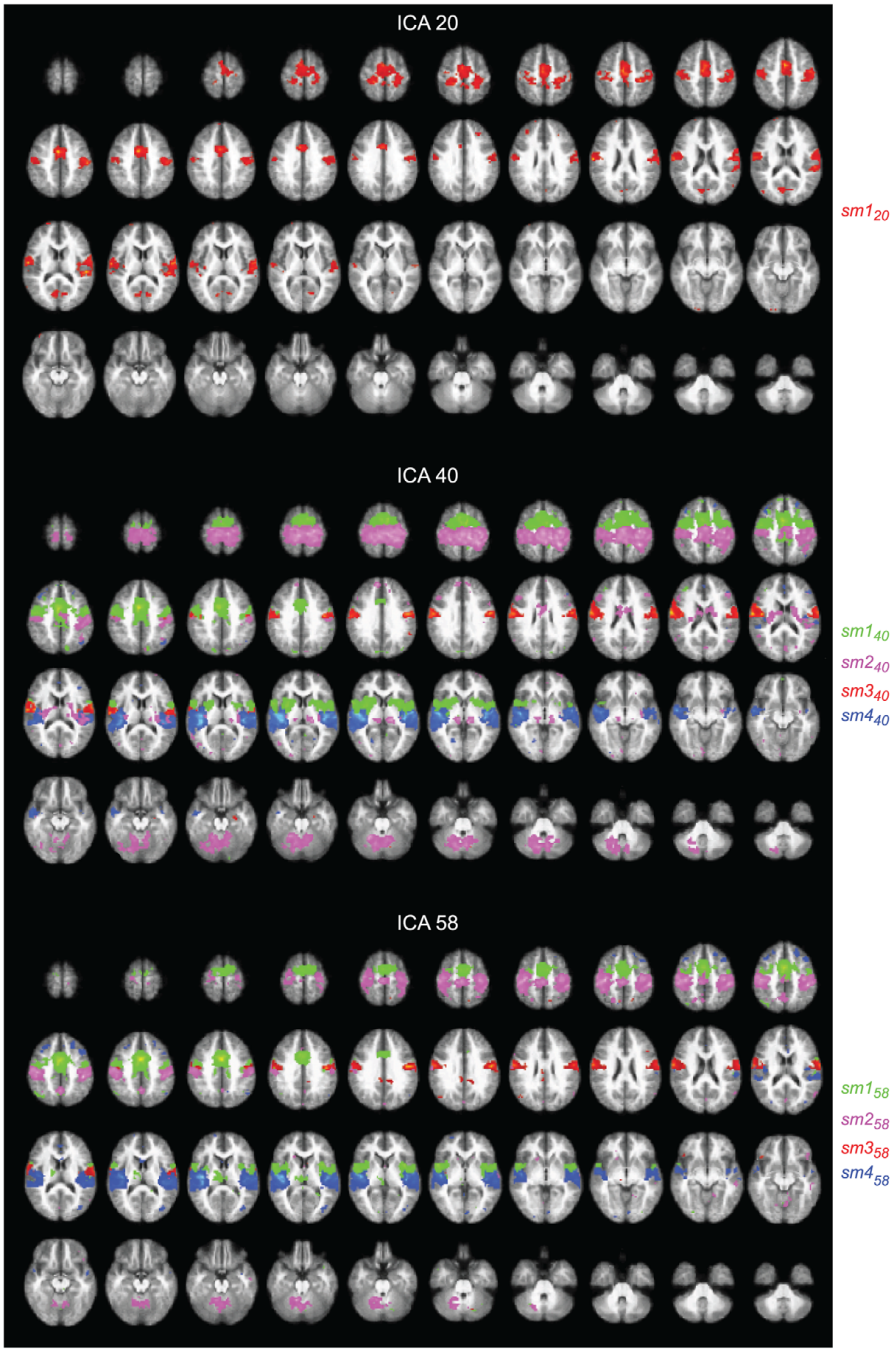
Sensorimotor network. Spatial maps for the ICs selected to represent the SMN in the 20- (top), 40- (middle), and 58-IC (bottom) decompositions, with 1, 4, and 4 color-coded components displayed, respectively. The maps are thresholded at *t ≥*6.

The following paragraphs and Figs. 2 and 3 explain the subdivision of the dorsal attention network and default-mode network in the 20-, 40-, and 58-IC decompositions. We will also describe (Fig. 4) the sensorimotor network SMN that was not visible in the 10-IC decomposition.

### Dorsal Attention Network

Figure 2 shows the ICs comprising the attention network in the 20-, 40-, and 58-IC decompositions.

#### 20-IC decomposition

In the 20-IC decomposition (top panels of Fig. 2), the IC1 network of the 10-IC decomposition (see Fig. 1) was covered by two components, *att1_20_*–comprising the conventional dorsal attention network and *att2_20_*–comprising mainly occipitotemporal visual cortices.

Component *att1_20_* (blue blobs in the top panels of Fig. 2) covers bilaterally the IPS, extending down to the supramarginal gyri, FEFs, and it also contains blobs in medial and posterior cingulate cortices, as well as in the posterior thalamus bilaterally.

Component *att2_20_* (red blobs in the top panels of Fig. 2) covers the most medial and posterior parts of the IPS in both hemispheres and FEFs bilaterally, largely overlapping the FEF activations of *att1_20_* but also extending anterior to them. Other nodes of this component include the fusiform cortices bilaterally, the calcarine sulcus, and the mid/superior occipital gyri.

#### 40-IC decomposition

In the 40-IC decomposition (Fig. 2, middle panels), four ICs were needed to cover the spatial maps of *att1_20_* and *att2_20_*, however in a more complex manner than by a simple division of each of the two components into distinct subcomponents. In contrast to the symmetric components in the 20-IC decomposition, *att1_40_* (blue blobs in the middle panels of Fig. 2) was now clearly left-lateralized and *att2_40_* (pink blobs in the middle panels of Fig. 2) right-lateralized. Notably, *att2_40_* included brain areas from both *att1_20_* and *att2_20_*.

Component *att1_40_* includes the left FEF, the left middle and inferior temporal areas, and the IPS bilaterally. It also covers parts of the cuneus and preC, the left thalamus, left hippocampus, and parts of the left mesial occipital cortex and left lingual gyrus.

Component *att2_40_* includes the right FEF, the right IPS, and the medioposterior part of the left IPS. It also comprises the mid/superior occipital gyri covered by *att2_20_*. Thus, this IC includes brain areas from both *att1_20_* and *att2_20_*.

Component *att3_40_* (red blobs in middle panels of Fig. 2) includes the inferior parts of *att1_20_*, that is bilaterally the lateral IPS and the supramarginal gyri and parts of the MCC. It also covers the posterior insulae of both hemispheres.

Component *att4_40_* (green blobs in middle panels of Fig. 2) covers bilaterally the fusiform cortices, the lingual gyri, as well as bilaterally the mesial occipital cortex (calcarine sulcus and middle occipital gyri), extending bilaterally to the superior parts of the cerebellum. This component also covers the left orbitofrontal cortex, as well as the putamen, caudate, and amygdala bilaterally.

#### 58-IC decomposition

The lowest panels of Fig. 2 show the 58-component decomposition, where two ICs were needed to cover the spatial map of *att1_20_* of the 20-IC composition: *att1_58_* (pink blobs in bottom panels of Fig. 2) the superior part (plus the inferior frontal gyri bilaterally) and *att2_58_* (green blobs in bottom panels of Fig. 2) the inferior part (plus the posterior insula bilaterally). These both components were rather symmetrical.

Component *att1_58_* covers bilaterally the superior IPS and the FEFs, and the right middle temporal areas. Component *att2_58_* covers the more lateral IPS down to middle temporal areas bilaterally. It also includes the precentral gyri and IPC in both hemispheres.

Component *att2_20_* of the 20-IC decomposition was split into three ICs in the 58-IC composition: *att3_58_*, *att4_58_*, and *att5_58_*. Component *att3_58_* (blue blobs in bottom panels of Fig. 2) comprises the left FEF, the left medial IPS, and, symmetrically but less extensively, the right IPS. It also includes the left fusiform and right amygdala, as well as the mid/superior occipital gyri bilaterally, and the left FFG. Component *att4_58_* (yellow blobs in bottom panels of Fig. 2) is lateralized to the right hemisphere, comprising the right FEF, the right medial IPS, and the right mid/superior occipital gyri. Component *att5_58_* (red blobs in bottom panels of Fig. 2) covers bilaterally the fusiform cortex, the mesial occipital cortex, and lingual gyri. It also includes the right FEF and the MCC.

### Default-mode Network

Figure 3 shows the spatial maps of the selected ICs for the DMN in the decompositions of 20, 40, and 58 components.

#### 20-IC decomposition

In the 20-IC decomposition, the DMN of the 10-IC decomposition was covered by one component *dm1_20_* very similar to that in the 10-IC but now including also the IPC in the right hemisphere. Furthermore, the prefrontal midline and inferior frontal activity appeared wider than in the 10-IC decomposition.

Component *dm1_20_* (red blobs in top panels of Fig. 3) covers parts of the posterior and middle cingulate cortices (PCC similarly and MCC more prominently than in the 10-IC decomposition), the preC, medial prefrontal cortex (MPFC), and bilaterally the IPC. It also extends bilaterally over the inferior frontal cortex.

#### 40-IC decomposition

In the 40-IC decomposition the DMN component of the 20-IC decomposition is split into *dm1_40_* (red blobs in middle panels of Fig. 3) concentrated on the MPFC and *dm2_40_* (blue blobs in middle panels of Fig. 3) covering the posterior parts of the DMN.

Component *dm1_40_* encompasses the MPFC as well as parts of the PCC and preC. It also includes a right-sided attention network (right FEF, right IPS, as well as left medial IPS), and it covers bilaterally the inferior frontal cortex as well as parts of the left occipital lobe.

Component *dm2_40_* demonstrates most prominent activity in bilateral IPC. It also includes the PCC and preC bilaterally and the left frontal cortex, as well as parts of the MCC and cuneus.

#### 58-IC decomposition

In the 58-IC decomposition, the component *dm1_20_* (of the 20-IC decomposition) was split into 5 subcomponents: the prefrontal cortex was split into one superior and one inferior component (*dm1_58_* and *dm2_58_*; yellow and blue blobs in bottom panels of Fig. 3), the bilateral IPC was covered by *dm3_58_* (pink blobs in bottom panels of Fig. 3), and the PCC/preC by *dm4_58_* (green blobs in bottom panels of Fig. 3). Moreover, the inferior parts of *dm1_20_* were now covered by component *dm5_58_* (red blobs in bottom panels of Fig. 3).

Figure 3 shows that *dm3_58_* includes the PCC, preC, MCC, and it also extends to the calcarine sulcus and lingual gyri. Component *dm4_58_* covers bilaterally the IPC as well as the MCC and parts of the left prefrontal cortex. Component *dm5_58_* encompasses bilaterally the inferior frontal gyrus and the left IPC (extending more inferior than component *dm4_58_*).

### Sensorimotor Network

Figure 4 shows the spatial maps of the selected ICs for the SMN in the decompositions of 20, 40, and 58 components; SMN did not appear in the 10-IC decomposition.

#### 20-IC decomposition

Component *sm1_20_* (red blobs in top panels of Fig. 4) corresponds to the SMN: it covers bilaterally the primary motor cortex (MI), the supplementary motor area (SMA), and the primary somatosensory cortices (SI) of both hemispheres. It also covers the Sylvian fissure, thereby overlapping with the secondary somatosensory (SII) and temporal-lobe auditory cortices in both hemispheres. In addition, it covers bilaterally parts of the mesial occipital cortex and medial cingulate cortex (MCC).

#### 40-IC decomposition

In the 40-IC decomposition, *sm1_20_* of the 20-IC decomposition was split into four subcomponents: *sm1_40_*, *sm2_40_*, *sm3_40_*, and *sm4_40_.* Component *sm1_40_* (green blobs in middle panels of Fig. 4) covers the SMA and premotor areas, down to the inferior SI. It also covers parts of the MCC and bilaterally the insula, putamen, and pallidum.

Component *sm2_40_* (pink blobs in middle panels of Fig. 4) is focused on the sensory areas but also covers MI, SII, and posterior SMA. It also encompasses superior parts of the cerebellum as well as bilaterally the insula, thalamus, and caudate.

The inferior parts the sensorimotor cortex are covered by *sm3_40_* (red blobs in middle panels of Fig. 4). The parieto-opercular (SII) cortex together with parts of the supratemporal auditory areas is covered by *sm4_40_* which also covers parts of the insula bilaterally (blue blobs in middle panels of Fig. 4).

#### 58-IC decomposition

In the 58-IC decomposition, four subcomponents–*sm1_58_*, *sm2_58_*, *sm3_58_*, and *sm4_58_*–cover the spatial map of *sm1_20_*. Component *sm1_58_* (green blobs in bottom panels of Fig. 4) is similar to *sm1_40_*, except that it does not reach the basal ganglia but instead covers the left thalamus. Component *sm2_58_* (pink blobs in bottom panels of Fig. 4) corresponds to *sm2_40_*, except that it includes the preC but not the insula, thalamus, or caudate. Component *sm3_58_* (red blobs in bottom panels of Fig. 4) is almost identical to *sm3_40_* and component *sm4_58_* (blue blobs in bottom panels of Fig. 4) corresponds to *sm4_40_*.

### Reliability of the Estimated ICs: ICASSO

**Figure 5:**
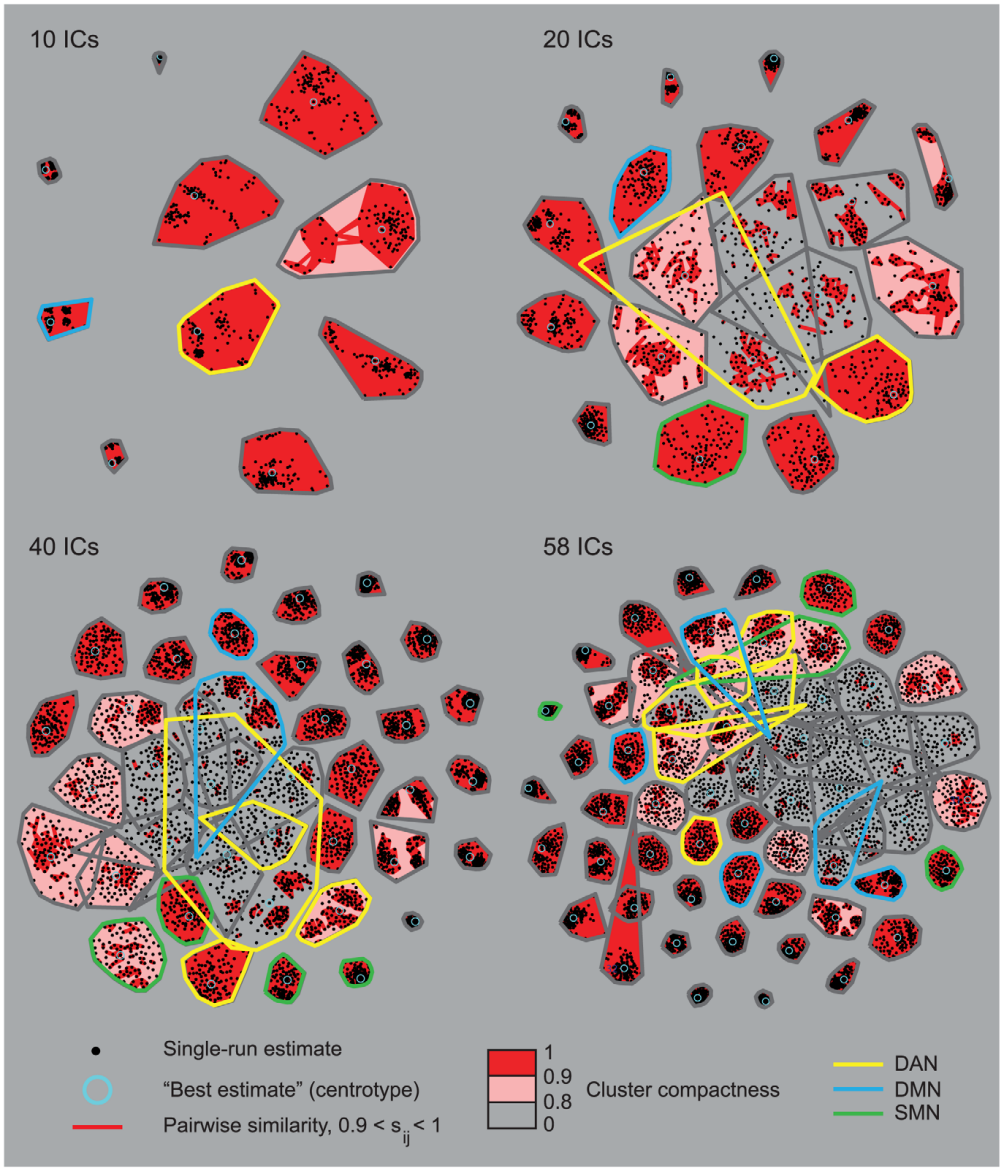
2D curvilinear component analysis projections of the clustered IC estimates. The 2D curvilinear component analysis projections of the clustered IC estimates for the 10-, 20-, 40-, and 58-IC decompositions. The black dots are the single-run-estimates of the ICs of each run of ICASSO. Each cluster refers to one IC. The pair-wise similarities s_ij_ inside each cluster are marked with red lines. Note that the pairwise similarities are not plotted if the average intra-cluster similarity is over 0.9. The best estimate (centrotype) of each cluster is circled with light blue. Cluster compactness (average intra-cluster similarity) is color-coded in 3 steps (0–0.8 gray, 0.8–0.9 pink, and 0.9–1 red). The ICs selected to represent the SMN, DAN, and DMN are marked with green, yellow, and blue outlines, respectively.

Figure 5 illustrates the 2D curvilinear component-analysis projections of the clustered ICASSO-based ICA estimates. The black dots represent the estimated components at every run of ICASSO. Small and tight clusters correspond to similar component estimates at every run. The average intra-cluster similarity is indicated by the background color. The ICs chosen to represent the DAN, DMN, and SMN are circled in Fig. 5 with yellow, blue, and green, respectively.

For the 10-IC decomposition, all components are stable and only one component has an intra-cluster similarity less than 0.9. At dimensionality of 20, most of the clusters are tight and well separated; four clusters have an intra-cluster similarity less than 0.8. From the chosen components, only *att1_20_* has an intra-cluster similarity lower than 0.8, whereas all other components have intra-cluster similarity over 0.9. The cluster corresponding to *att1_20_* is wide and overlaps with other clusters, meaning that the component estimates vary considerably between different runs.

When the dimensionality is increased to 40, some of the clusters start to overlap and the intra-cluster similarity is less than 0.8 for nine clusters; these clusters include two ICs of the DAN (*att2_40_* and *att4_40_*) and one IC of the DMN (*dm2_40_*).

At dimensionality of 58, many clusters are still tight and well separated, but some of them in the center of the graph overlap considerably, and several clusters have an intra-cluster similarity lower than 0.8. Thus the ICA estimation starts to become less stable at high dimensionalities. One component of the DAN (*att1_58_*) and one component of the DMN (*dm1_58_*) are unstable with intra-cluster similarity less than 0.8.

## Discussion

We analyzed three major cortical networks, *i.e.* the dorsal attention, default-mode, and sensorimotor networks, extracted from fMRI data acquired from healthy young adults who were freely viewing a silent film. The main aim was to examine how the network division changes as a function of the number of components (10, 20, 40, or 58) in the independent component analysis. The highest dimension, 58 components, was proposed by the minimum-description-length algorithm, which has the property of statistical consistency and thus yields asymptotically correct results [16], [17]. We expected the subdivision of the components at higher dimensionalities (40, 58) to tell about the functional parcellation of the underlying circuitry.

As expected, the dorsal attention network, default-mode network, and sensorimotor network were fragmented into smaller sub-networks as the dimensionality of the IC-decomposition increased. Unexpectedly, the new higher-dimension decomposition of 40 components included a component that merged brain areas from more than one component of the lower dimension decomposition, indicating that we were not only witnessing subdivision of the same components. This finding likely reflects the highly similar activation time courses of these areas accompanied by the proximity of the areas.

Previously, the dorsal attention, default-mode, and sensorimotor networks have been characterized on the basis of resting-state fMRI data [18]–[21], implying that the nodes of these networks are functionally connected even in the absence of external stimuli. However, some connections may strengthen and others weaken in task conditions, as we expected to happen when our subjects viewed a visually rich movie stimulus. Notably, free movie viewing involves attentive monitoring of the visual scenes and socially relevant events on the screen. Previous studies have shown that a well-directed movie guides different viewers’ attention–as reflected by eye-gaze patterns–much more similarly than does, for example, a surveillance camera video from a vivid city square [22]. The experimental film ‘At Land’ presented in the current study depicts often surprising social and physical events that maintain the subjects’ attention during the whole 15-min movie. Thus, a close coupling between the dorsal attention network and visual areas, including face- and body-sensitive fusiform areas, was to be expected and such connection was observed in the low-dimensional decompositions where the visual and attention networks belonged to the same components.

In the following we discuss some functionally interesting findings.

### Splitting of the IC Networks

#### 10-IC decomposition

Our 10-IC decomposition was of far too low dimensionality according to all standards of IC- number selection. Although DAN and DMN were clearly evident in distinct components, these components also comprised brain areas that are not typically considered to belong to these networks. Two of the ten ICs were clear artifacts.

It is possible although quite unexpected that the DMN was split into two components already at this dimensionality (ICs 2 and 9), although the parietal nodes of IC 9 were located somewhat posterior to the brain areas typically considered to belong to the DMN [21]. DMN activity is known to decrease during task performance [21], and watching the movie may have weakened the connections between the DMN nodes, resulting in the splitting already at this low dimensionality.

As the movie stimulus included significant amount of body movements, such as grasping hands, we expected the SMN to appear in all IC dimensionalities. However, the rather prominent SMN at other dimensionalities was not yet visible at the 10-IC decomposition. One possible explanation is that clear SMN signal appeared only in some subjects and thus when only 10 components were extracted, the SMN did not survive the final reduction step of ICA. Another possibility is that the variability in inter-node connectivity was higher for the SMN than for the now detected 10 components. Moreover, the signal may have varied more across subjects for the SMN than for the other networks, or the SMN may have been obscured by noise at this low dimensionality.

These results imply that the most task-related brain networks are revealed already at a very low dimensionality of ICA.

#### Dorsal attention network

The now already classical dorsal attention network is related to voluntary orienting towards stimuli [23]. When identified during resting state, the DAN comprises, rather symmetrically, the lateral intraparietal cortices, FEFs, and visual cortices, especially MT/V5 region. Our DAN also included, at low (10 and 20) dimensionalities, the fusiform face region. We interpret this close connection of a wider visual network and the DAN to be due to the movie stimulus where social stimuli, especially close-ups of faces, were strong attention capturers.

When the dimensionality was increased from 10 to 20 components, the superior parts of the DAN detached from the inferior network and visual areas. The superior DAN component seemed to behave more like a top-down attention network, without coupling to the visual areas.

Some of the brain areas of the DAN covered by two distinct ICs in the 20-IC decomposition merged into one IC in the 40-IC decomposition, implying relatedness of the two ICs of the 20-IC decomposition. Interesting hemispheric lateralization also emerged, as one FEF–IPS subcomponent was lateralized to the right hemisphere but also included the medial aspects of the left IPS, whereas a corresponding left-hemisphere FEF–IPS component only covered IPS in the left hemisphere.

Several studies have shown right hemisphere dominance for spatial attention. For example, lesions in the right temporoparietal junction or parietal lobe cause a more severe contralesional neglect than do equivalent lesions in the left hemisphere [24]–[29]. Accordingly, fMRI studies have demonstrated stronger activation in the right than left parietal lobe in attention shifting tasks [30], [31]. Thus the DAN subcomponents in the 40-decomposition agree with the well-established role of the right hemisphere in directing attention to both hemispaces whereas the left hemisphere shifts attention predominantly in the contralateral hemispace [32]. In other words, the network seemed to fragment into functionally feasible subunits.

It was also interesting to note that the partly overlapping components of 20- and 58-IC decompositions covered slightly different areas of the FEF. FEF is known to have subdivisions preferentially representing the central and peripheral visual fields. For example, Gitelman and coworkers [33], contrasting exploration and central fixation, noted that the FEF region for visual search is medial to that of oculomotor control and the area for covert attention was in-between, with partial overlap. Compared with these studies, our present ICA results are not based on contrasts between different task conditions but rather directly capture functionally connected networks during the whole movie stimulus, thereby providing further support for the functional parcellation of the IPS–FEF network.

Different parts of the lateral intraparietal sulcus (LIP) have different roles in oculomotor control and attention, whereas the anterior intraparietal sulcus (AIP) is connected to eye fixation and to manipulation and grasping of objects [34]. It is possible that a large part of the prominent IPS activation that we detected in the present study was related to saccades during free viewing of the movie. Posterior IPS, corresponding to our most medial IPS, has also been claimed to have a role in object-centered social orienting [35], and its activation in the present study could have received contribution from processes related to anticipation of object-oriented actions of the main character in the movie.

#### Default-mode network

At the dimensionality of 40 components, the DMN was split into two subcomponents, the other concentrated on the MPFC and the other on the more posterior nodes of the DMN. Previous studies support the idea of these two subsystems in the DMN [36]. The posterior subsystem has been linked for example to episodic memory retrieval [37], whereas the anterior subsystem is activated during self-referential thinking [38].

In the 58-IC decomposition, the MPFC was split into two DMN components, one covering the ventral and the other the dorsal part of the MPFC. According to previous imaging studies, the dorsal MPFC is involved in affective and emotional processing, whereas the ventral MPFC is activated during attention-demanding cognitive processing (for reviews, see [39]–[41]). For example, self-referential tasks were associated with activity increases in the dorsal MPFC while the activity in the ventral MPFC decreased [38].

Solely the preC/PCC formed one subcomponent of the DMN in the 58-IC decomposition. This area seems to be a key node in the DMN, connecting information from the subsystems of the network [42]. Leech and collaborators [43] recently demonstrated functional subdivision of the PCC to subregions that have connections to several other cortical circuits beyond the DMN. PCC was assumed to act as a hub integrating information from several cortical networks. Importantly, only the ventral part of the PCC showed functional connectivity with the other nodes of the DMN, whereas other parts were connected with e.g. dorsal attention network or sensorimotor areas. Our results are well in line with these Leech et al. [43] findings, as some PCC regions were included in the SNM- and DAN-related ICs.

#### Sensorimotor network

We selected a visual stimulus, which displayed abundant sensorimotor features, such as crawling, climbing, manipulation of small chess pieces, striking another person’s hair, and collecting rocks, to deliberately activate the viewer’s SMN network. As mentioned above, the SMN was not visible at the dimensionality of 10 components, but it appeared when the dimensionality was increased to 20 components.

At the rather low dimensionality of 20 ICs, the sensorimotor IC included superior temporal lobes and thereby also auditory areas. The SII and parts of the auditory cortex occurred in same IC also when the dimensionality of ICA was increased. As the film stimulus was silent (apart from noise of the fMRI scanner itself), this finding might not reflect auditory-cortex activation but just be a methodological artifact due to the unknown spatial extent of any fMRI activation; the extent is always defined by statistical thresholding. The secondary somatosensory cortex in the parietal operculum and the supratemporal auditory cortex are close to each other on the opposite walls of the Sylvian fissure. Albeit, we cannot rule out a real auditory-cortex activation because of multisensory integration, caused by visually perceived sources of sounds, such as the ocean waves crashing to the shore, seagull sounds, or dinner-table conversation that may activate the auditory cortex during viewing of the silent movie [44].

The SMN was split into functionally feasible subnetworks when the dimensionality of ICA was increased to 40 components but it did not split further when the dimensionality was increased to 58 components. Similarly as here, Abou-Elseoud and coworkers [5] noticed that some components did not split any further when the dimensionality of ICA was increased. They suggested that the stable components might represent less connected nodes, while the branching ones are kind of connector hubs with lots of connections to other nodes. Consequently, the stable components, with fewer connections, are functionally more independent.

### Reliability of the IC Estimation in ICASSO Analysis

ICASSO indicated decreased repeatability of the ICs when the number of component estimates was increased; with 58 ICs, a large portion of the clusters of the single-run-estimates overlapped considerably and the ICA estimation was less stable at a high dimensionality. Significant reduction in ICA repeatability with increasing model order has been reported earlier [5], [7].

The most stable components included e.g. the early visual areas and the insula whereas the selected ICs representing the three networks were less stable. One apparent explanation is that activity in the higher-order complex networks varies more across subjects than does processing in e.g. sensory projection cortices.

#### Relating the findings to film features

A movie is a rich and complex stimulus that captures the viewers’ attention in a totally different manner than simple (and static) stimuli do. Thus it would be interesting to relate the networks and their subnetworks to film features to see whether the hierarchy of the networks could be explained by differential brain processing. Our preliminary results from an on-going study show that the mean rating (obtained from 14 subjects separate from the current study group) of the amount of tactile experience during the movie viewing correlates well with one sensorimotor IC of the 40-IC decomposition as well as with the ICs of the dorsal attention network at all dimensionalities. Collecting such ratings of different movie features–for example of the amount of faces, social interaction, biological motion, or the valence and arousal [45]–[47]–would help to unravel the effect of movies on various brain networks.

### Conclusions

We applied group ICA to fMRI data that had been acquired during free viewing of a silent short film. As expected, when the component number increased from 10 to 20, 40, and 58, the IC’s provided more detailed information of the functionally specific subnetworks of the three major networks (DAN, DMN, and SMN). Although DAN and DMN were clearly evident already at the lowest dimensionality, they also comprised brain areas that are not typically considered to belong to them.

The major networks were fragmented into functionally feasible components when the number of estimated ICs was increased. It thereby seems tenable that running the ICA from the same fMRI data with different numbers of the estimated ICs can unravel parcellation of the total brain activity into functionally meaningful subnetworks.

Our results also contribute to the now growing evidence that fMRI activity collected during movie viewing can, despite of its complexity, be analyzed in a reliable manner and that the results are consistent. Application of naturalistic stimuli during functional brain imaging opens up new possibilities for future studies of the brain basis of several cognitive functions.

## Materials and Methods

### Subjects

Twenty-two healthy adults participated after written informed consent. The study had a prior approval by the Ethics Committee of Helsinki and Uusimaa Hospital District. The data of 7 subjects were rejected because of technical problems, drowsiness, or excessive head movements, and thus the following analyses are based on data of 15 subjects (9 females, 6 males; mean age 24 years, range 19–49).

### Stimuli

During the fMRI scanning, the subjects were viewing a silent black-and-white film (“At Land” by Maya Deren, 1944). In the film a determined-looking female character is searching for something in different environments. In addition to social interaction situations, the film features a range of sensorimotor activities, such as climbing, crawling, jumping, falling, as well as manipulation of small objects. The film was delivered using the Presentation software (version 0.81, http://www.neurobehavioralsystems.com) and projected (projector Vista X3 REV Q, Christie Digital Systems, Canada, Inc.) to a semitransparent back-projection screen that the subjects viewed via a mirror (visual angle 36° horizontal, 29° vertical).

### Data Acquisition

The fMRI images were acquired with a Sigma VH/I 3.0 T MRI scanner (General Electric, Milwaukee, WI, USA). Functional images were obtained using a gradient echo-planar-imaging sequence with following parameters: TR 2.015 s, TE 32 ms, FA 75°, 34 oblique axial slices, slice thickness 4 mm, matrix 64×64, voxel size 3.4×3.4×4 mm^3^, field of view (FOV) 22 cm. Altogether 485 volumes were collected per subject, including 4 dummy scans that were removed from further analysis. Structural images were scanned with 3-D T1 spoiled gradient imaging, matrix 256×256, TR 10 ms, TE 3 s, flip angle 15°, preparation time 300 ms, FOV 25.6 cm, slice thickness 1 mm, number of excitations 1.

### Pre-processing

The fMRI data were preprocessed using SPM8 software (http://www.fil.ion.ucl.ac.uk/spm/software/spm8/), including realignment, co-registration, normalization into MNI space, and smoothing with a 6-mm (full-width-at-half-maximum) Gaussian filter. Before normalization, the images were skull-stripped using the FreeSurfer software (http://surfer.nmr.mgh.harvard.edu/).

### Independent-component Analysis

The IC analysis was performed with the GIFT software (version v2.0e, http://icatb.sourceforge.net/groupica.htm) for group-ICA [48]. The minimum-description-length-based estimation, implemented in GIFT, suggested 58 ICs. We thus decided to estimate 10, 20, 40, and 58 ICs with Fast ICA algorithm. We used the back-reconstruction method GICA3 that has been shown to provide robust and accurate results [49]; see also Appendix S1 for our arguments of choosing GICA3. ICASSO [50] analysis was performed to examine the reliability of the IC estimates.

For further analysis, we employed the across-subjects *t*-maps computed by GIFT, thresholded at *t* ≥6. All ICs of the 10-IC decomposition were included in the final analysis. From the 20-IC decomposition, 2 ICs were selected by visual inspection to represent and cover the dorsal attention network DAN, 1 IC to cover the default-mode network DMN, and 1 IC to cover the sensorimotor network SMN. From the 40-IC and 58-IC decompositions, ICs were selected by visual inspection so that they together resembled the spatial maps of the selected ICs from the 20-IC decomposition; this procedure resulted in 2–5 ICs per network. To facilitate the selection, we spatially correlated the maps from the 40- and 58-IC decompositions with the respective 20-component maps.

## Supporting Information

Appendix S1 (DOC)
