# Supplementary material for: Functional Subdivision of Group-ICA Results of fMRI Data Collected during Cinema Viewing
Source: PLoS One. 2012 Jul 30;7(7):e42000. doi: 10.1371/journal.pone.0042000 (PMC3408398; doi:10.1371/journal.pone.0042000)
Supplement: Appendix S1 — (DOC) [file pone.0042000.s001.doc]

# Appendix S1

In this appendix we note some short-comings of the original back-reconstruction method GICA1 of Group ICA, and of the original derivation of GICA3 method. We finish by showing why GICA3 is nevertheless the method of choice.

## GIGA1

As becomes apparent below, the original [48] and currently the default back-reconstruction method in GIFT (version 2.0e), nowadays called GICA1 [49], is only an approximation. Using the notation in [48], the derivation from Equation A1 to A2 in that article (even considering errata [51]) should be:

(A.1)

(A.2)

(A.3)

where the matrices are as in the original article and the main difference is $GG^{-1}$ in Equation A.2 and the approximation sign in Equation A.3 above. Specifically, $G^{-1}$ marks the pseudo-inverse of $G$ in that article, and $G^{-1}$ is the N-by-LM group reducing matrix, where N is the number of independent components (ICs), L is the size of the per-subject time dimension following the per-subject PCA reduction, and M is the number of subjects. Since $N<LM$, the matrices $G$ and $G^{-1}$ have (maximum) rank of N. Thus the N-by-N matrix $G^{-1}G=I$ and the LM-by-LM matrix $GG^{-1}\neq I$ (see Equation A.2 above), where $I$ is the identity matrix. Therefore, all equations A2−A6 in [48] are approximations only.

## PCA vs. Whitening

The data reduction step (Material and Methods, PCA) in [49] describes the use of orthonormal eigenvectors of the PCA for data reduction and derives the equation $F^{-}=F^{T}$ from Equation 1 of that article (the article uses notation $F^{-}$ for the pseudo-inverse of $F$). Whereas this equation is correct when using the definitions in that article (i.e. $F$ contains only orthonormal vectors), the equation does not hold for whitening matrices used for ICA in general and as implemented within GIFT's Matlab code (current version 2.0e).

For whitening and dewhitening operators

$(FD^{1/2})^{-}=D^{-1/2}F^{T}\neq D^{1/2}F^{T}=(FD^{1/2})^{T}$ (A.4)

where $D$ is the diagonal matrix containing eigenvalues as stated in Equation 1 of [49]. Therefore, all equations of [49] using assumption $F^{-}=F^{T}$ are incorrect when $F$ is a whitening or dewhitening matrix. For example, when the group reducing matrix $G^{-}$ is a whitening matrix (i.e. as is implemented in GIFT Matlab code) the Equation 3 of that article has $G^{-}\neq[G_{1}^{T},\ldots,G_{M}^{T}]$. Respectively for GICA3 derivation, the Equation 8 of the article has

$R_{i}=F_{i}(\{G^{-}\}_{i})^{-}A\neq F_{i}(G_{i}^{T})^{-}A$ (A.5)

where $\{G^{-}\}_{i}$ is a partitioning of the whitening matrix $G^{-}$ and $\{G^{-}\}_{i}\neq G_{i}^{T}$.

## GICA3

Despite of the short-comings in the development of GICA3 mentioned above, we now show that it actually is the mathematically valid back-reconstruction method in GIFT. The derivation can be made straight from the basic equations: Equation A1 in [48], Equation 3 in [49], or Equation A.1 in this Appendix. In the following, we use notation $G^{-}$ to mark the pseudo-inverse of $G$, and no assumptions are made about the reduction matrices $F_{i}^{-}$ and $G^{-}$. They can be any reduction matrices including whitening matrices, orthonormal projections, and other. Specifically, $F_{i}^{-}\neq F_{i}^{T}$ and $G^{-}\neq G^{T}$ may hold.

Marking $G^{-}=H=[H_{1},\ldots,H_{M}]$ for clarity (i.e. $H_{i}$ is a partition of the pseudo-inverse of $G$, in practise a partition of the whitening matrix $G^{-}$) we get

(A.6)

$=\sum_{i=1}^{M} H_{i}F_{i}^{-}Y_{i}$ (A.7)

(A.8)

$S_{i}=\hat{A}^{-}H_{i}F_{i}^{-}Y_{i}$ (A.9)

As can be seen, Equation A.9 above is identical to Equation 12 in [49] if matrices $G$ and $F_{i}$ contain orthonormal vectors only and thereby $H=G^{-}=G^{T}$ and $F_{i}^{-}=F_{i}^{T}$ would hold. In practise, GICA3 algorithm in GIFT's Matlab code (v2.0e) is implemented by taking partitioning of the whitening matrix, i.e. implemented correctly considering our Equation A.9 above. Fortunately, the actual implementation for the GICA3 back-reconstruction routine does not follow Equation 12 in [49] and use the transpose (or pseudo-inverse) of the partitioning ($G_{i}$) of the dewhitening matrix $G$. Thus, GICA3 implementation in GIFT produces mathematically valid decomposition with $\hat{S}=\sum_{i=1}^{M} S_{i}$ and is therefore our method of choice.

**Supporting Reference**

51. Calhoun VD, Adali T, Pearlson GD, Pekar JJ (2002) A method for making group inferences from functional MRI data using independent component analysis. Hum Brain Mapp 16: 131–131.
